# Hepatic Epithelioid Hemangioendothelioma With Unique Metastasis to the Superior Vena Cava Found on CT Imaging: A Case Study

**DOI:** 10.7759/cureus.26155

**Published:** 2022-06-21

**Authors:** Charles A Bisbee, Logan Burstiner, Hristina Natcheva

**Affiliations:** 1 Radiology, Nova Southeastern University Kiran C. Patel College of Osteopathic Medicine, Davie, USA; 2 Radiology, University of South Florida, Tampa, USA

**Keywords:** vascular malignancy, metasasis, liver transplantation, superior vena cava, hepatic epithelioid hemangioendothelioma

## Abstract

Hepatic epithelioid hemangioendothelioma (HEHE) is a rare malignant tumor of vascular origin. Classically, HEHE is typically associated with imaging demonstrating multifocal heterogeneously enhancing hepatic nodules and histologic examination revealing mixed epithelioid and dendritic cells in a proliferative fibrous stromal background. While generally described as a low-to-intermediate grade indolent tumor, it is essential to establish the presence or absence of extrahepatic spread when considering transplant candidacy. We describe one case study in which a transplant was denied to a unique metastatic pattern of HEHE to the superior vena cava. This is a previously unreported location of metastasis of HEHE and may serve to broaden our current understanding of potential metastatic sites for this disease.

## Introduction

Hepatic epithelioid hemangioendothelioma (HEHE) is a rare malignant tumor of vascular origin with an estimated annual incidence of less than 0.1 per 100,000 [1]. The disease most commonly presents between ages 30 and 50, with a predilection for females over males [1]. Contact with vinyl chloride and oral contraceptive use has been suggested to increase the risk for the development of the disease [1]. HEHE is discovered incidentally in many cases, with over a quarter of patients experiencing no symptoms. When symptomatic, most patients present with abdominal pain, weight loss, or ascites [1,2]. HEHE is typically added to the differential when imaging demonstrates multifocal heterogeneously enhancing hepatic nodules [3]. Histologic examination classically reveals mixed epithelioid and dendritic cells in a proliferative fibrous stromal background. The similar appearance of more common liver lesions such as hepatocellular carcinoma, angiosarcoma, metastatic carcinoma, and cholangiocarcinoma frequently leads to misdiagnosis. However, HEHE can be differentiated by its immunohistochemical expression of endothelial markers and lack of epithelial markers [1,4].

No standard treatment guidelines or protocols currently exist. In many cases, management involves a variable combination of surgical resection, liver transplant, and chemotherapy [2]. While generally described as a low-to-intermediate grade indolent tumor, it is essential to establish the presence or absence of extrahepatic spread when considering transplant candidacy. The most commonly involved sites include the lungs, local lymph nodes, peritoneum, bone, spleen, and diaphragm [5]. Below, we discuss a case of HEHE in a 31-year-old female with a unique pattern of metastasis to the superior vena cava.

## Case presentation

**Figure 1:**
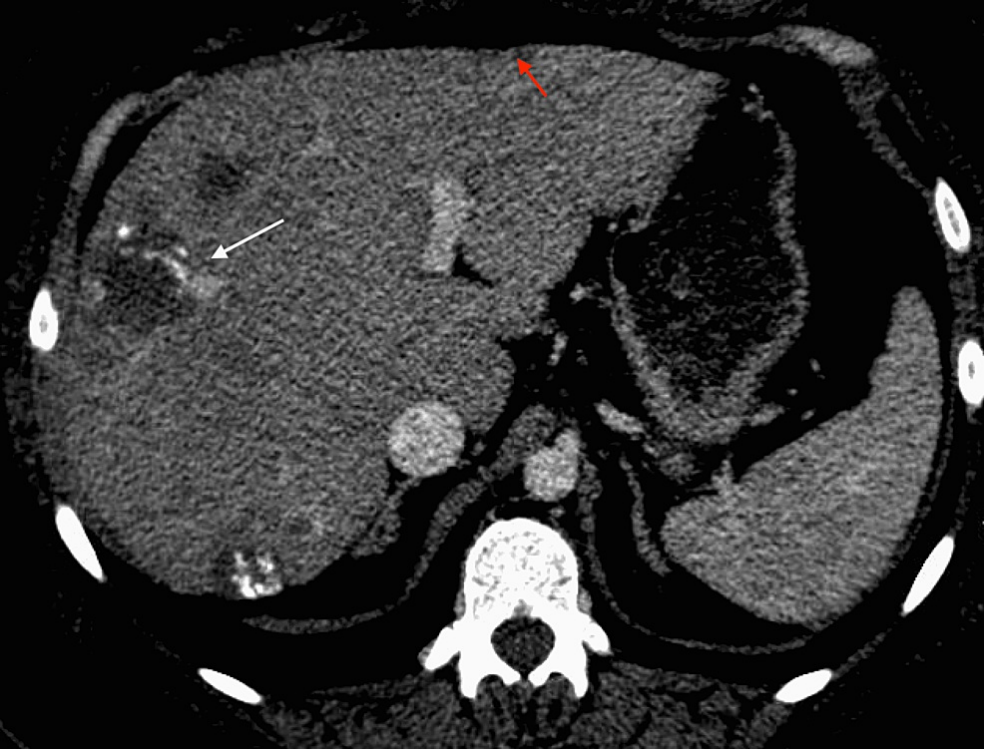
Axial view of a CT abdomen/pelvis showing capsular retraction (red arrow) as well as the “lollipop sign” (white arrow) CT: Computed Tomography

**Figure 2:**
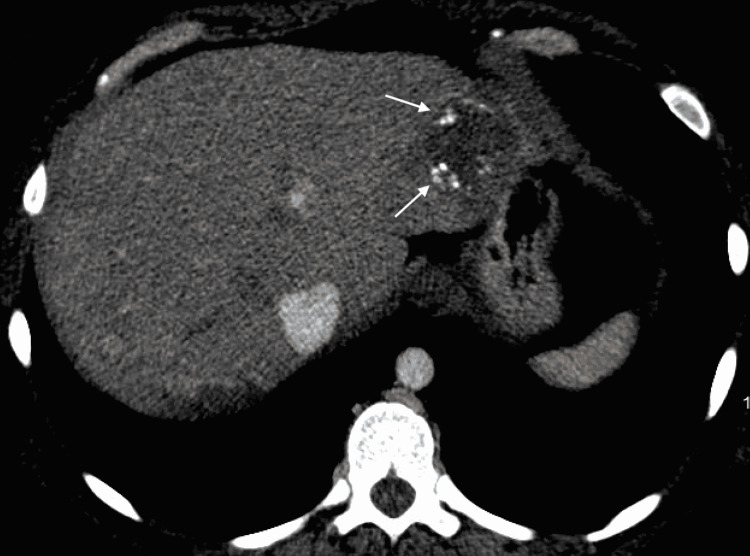
Axial view of a CT abdomen/pelvis showing a hepatic lesion with multiple calcifications present (white arrows) CT: Computed Tomography

**Figure 3:**
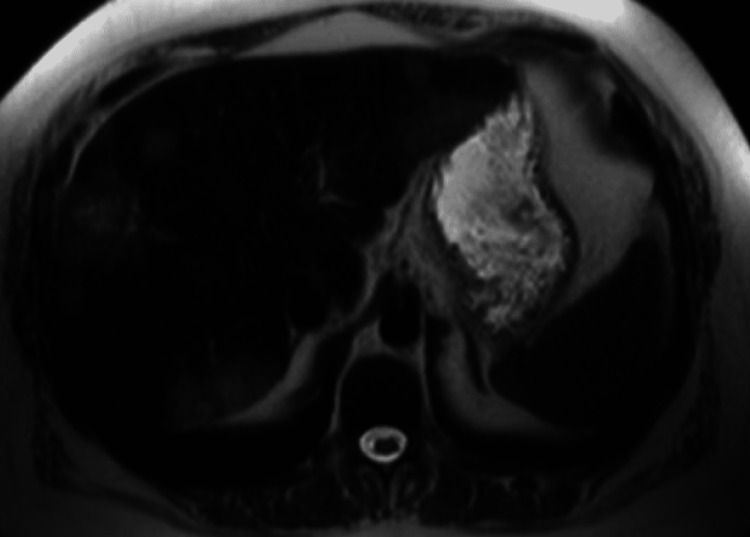
Axial view of an MRI abdomen T-2 weighted imaging showing numerous hyperintense hepatic lesions MRI: Magnetic Resonance Imaging

**Figure 4:**
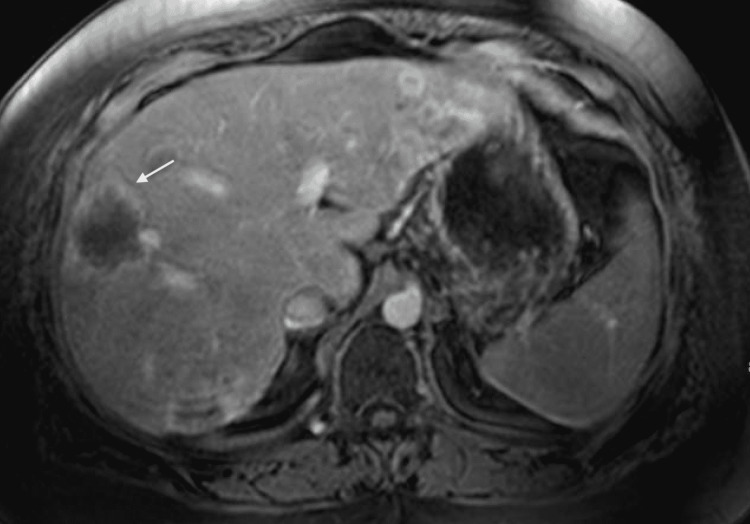
Axial view of an MRI abdomen T-1 weighted imaging taken one minute post contrast showing a hepatic lesion with peripheral enhancement (white arrow) MRI: Magnetic Resonance Imaging

Our patient is a 31-year-old female with type two diabetes mellitus who presented to the hospital with abdominal pain, nausea, and vomiting. She was diagnosed and admitted for diabetic ketoacidosis and pancreatitis. Along with inflammation of the pancreas, computed tomography (CT) of her abdomen and pelvis revealed numerous peripherally hyperattenuating targetoid lesions throughout the liver as well as capsular retraction, numerous calcifications, and the “lollipop sign” indicating hepatic vein branches tapering at the edge of a lesion with an avascular core (Figures 1, 2). Of these findings, the “lollipop sign” improves the specificity of a diagnosis of HEHE as none of the differential diagnoses for the disease exhibit a similar pattern of venous involvement [6]. Subsequent MRI further demonstrated multiple hyperintense hepatic lesions that showed peripheral enhancement consistent with the pattern described on CT (Figures 3, 4). Initially thought to be a metastatic disease versus abscesses, histological examination after CT-guided liver biopsy demonstrated prominent hyalinized stroma and scattered epithelioid cells with vacuoles present. Immunohistochemical staining was positive for CD31, CD34, and CK7 and weakly positive for cytokeratin AE1/3 and Cam5.2, compatible with HEHE.

**Figure 5:**
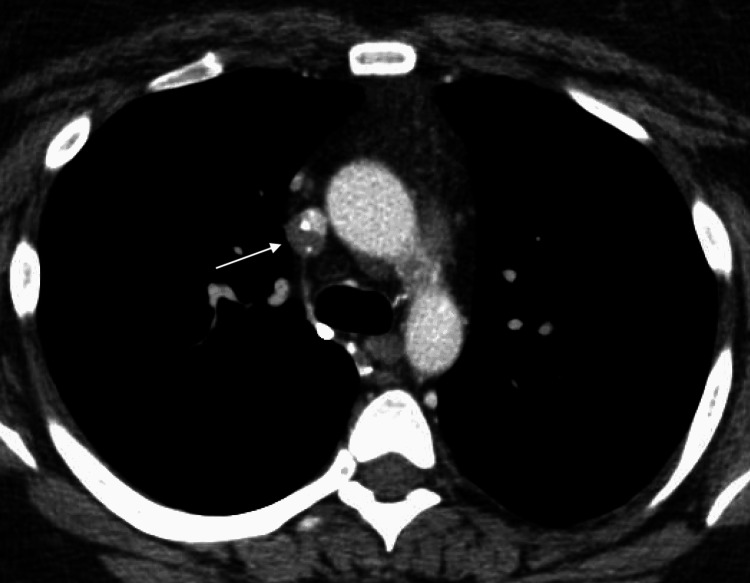
Axial view of a CT chest showing an eccentric, partially calcified, hypodense nodule (1.1 x 1.4 cm, white arrow) abutting and encroaching into the lower SVC CT: Computed Tomography, SVC: Superior Vena Cava

**Figure 6:**
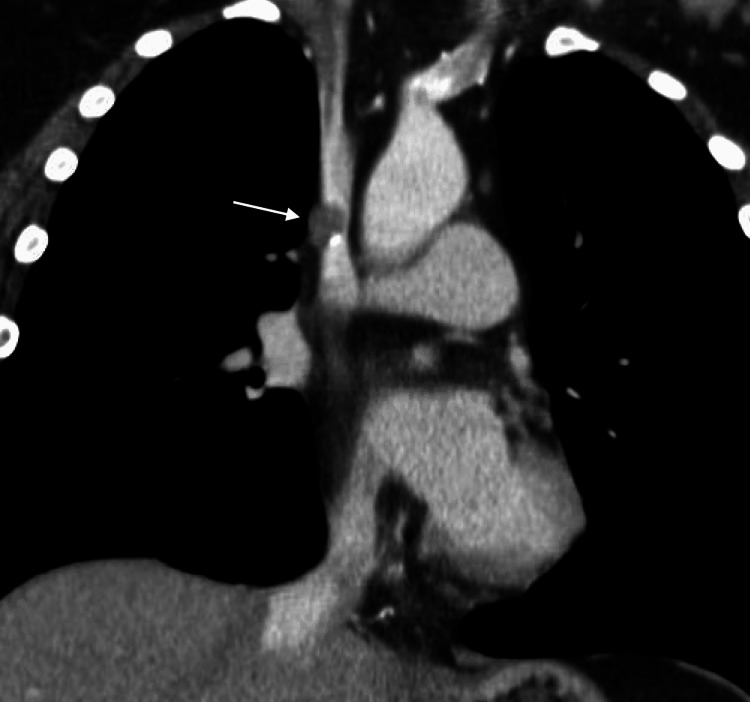
Coronal view of a CT chest showing another view of the eccentric, partially calcified, hypodense nodule (1.1 x 1.4 cm, white arrow) abutting and encroaching into the lower SVC CT: Computed Tomography, SVC: Superior Vena Cava

Initial workup, including MRI and PET/CT, were negative for metastatic disease, and she was referred for liver transplant evaluation. Unfortunately, during her subsequent liver transplant evaluation, CT of the chest revealed an eccentric, non-occlusive, partially calcified hypodense nodule abutting and encroaching the lower superior vena cava (Figures 5, 6). Under fluoroscopic and intravascular ultrasound guidance, biopsies of the lesion were obtained. Pathologic examination confirmed epithelioid hemangioendothelioma, excluding her from transplant candidacy. She was referred to an outside institution for a possible clinical trial and subsequently lost to follow-up.

## Discussion

As in our case, HEHE is often initially mistaken for metastases to the liver due to its multifocal appearance [7]. In turn, due to there being less than 500 reported cases, information delineating the specific metastatic patterns of HEHE is limited. In a review of 402 cases of HEHE from 1984 to 2005, 63.4% of patients had no evidence of extrahepatic disease. When present, lesions were most commonly found in the lungs, followed by regional lymph nodes, peritoneum, bone, spleen, and diaphragm, in that order [8]. Other researchers have noted that HEHE most commonly tends to spread locally, commonly involving the peritoneum, gut, and lungs [9,10]. Spread to the superior vena cava, as seen in our patient, is a previously unreported occurrence. In contrast to HEHE, extrahepatic primary epithelioid hemangioendothelioma is more common and is known to involve multiple areas of the body, with distant metastases to the lungs, mediastinum, thyroid, peritoneum, lymph nodes, bone, palate, and liver. Interestingly, cases of primary SVC epithelioid hemangioendothelioma have been reported. Though most of these patients are minimally symptomatic despite imaging findings of occlusion, the disease can progress to cause rapid onset superior vena cava syndrome [11,12]. 

No standard HEHE treatment guidelines exist. Variable combinations of partial hepatectomy, liver transplantation, and radiotherapy/chemotherapy have all been explored [5]. Additionally, in cases where concern for progression was low, observation and waiting have been tried. In one review, liver transplantation was the most common intervention (44.8% of cases), followed by non-treatment (24.8%), chemotherapy/radiotherapy (21%), and partial hepatectomy (9.4%). For cases of HEHE with small, isolated lesions, partial resection has favorable results. Conversely, transplantation is preferred with multifocal disease [5]. A study by Liu, however, showed that despite surgical intervention recurrence of HEHE remained high, and many patients with HEHE had a high chance of progressive disease [13]. For primary epithelioid hemangioendothelioma of the superior vena cava, as well as in metastasis to the SVC in which occlusion is a concern, surgical resection would be the optimal approach [11]. For diagnosis, contrast-enhanced CT, conventional venography, and PET-CT are useful and can help guide surgical intervention. Overall, despite a lack of concrete guidelines regarding management, HEHE has a relatively good prognosis compared to other malignant tumors of the liver, with one study observing that metastasis did not appear to shorten the survival rate [14]. Other studies, however, have found interventions such as hepatectomy and liver transplantation to promote long-term survival [8,15]. Regardless, the presence of extrahepatic malignancy or metastasis, as seen in our patient, remains an absolute contraindication for liver transplantation [16].

## Conclusions

HEHE is a rare entity that should remain in the differential diagnosis of any patient with heterogeneously enhancing liver nodules on imaging. Knowing the various radiologic findings of the disease, as well as common metastatic patterns can help in differentiating this tumor from other hepatic masses, which may lead to timely and cost-effective care for the patient. While more common sites of metastasis have been identified, the disease has shown the ability to metastasize to unique locations such as the superior vena cava, and radiologists should maintain a high degree of suspicion for unconventional metastatic patterns. With no standard guidelines for treatment currently existing, further research may be helpful in determining the best approach to management. In patients undergoing transplants, it is essential to identify potential metastatic sites which may serve as an absolute contraindication to the procedure, further emphasizing the importance of understanding potential metastatic locations.
